# Fixed Versus Variable Dosing of Prothrombin Complex Concentrate in Vitamin K Antagonist-Related Intracranial Hemorrhage: A Retrospective Analysis

**DOI:** 10.1007/s12028-016-0248-8

**Published:** 2016-04-06

**Authors:** Rahat Amadkhan Abdoellakhan, Ishita Parveen Miah, Nakisa Khorsand, Karina Meijer, Korné Jellema

**Affiliations:** 1 Department of Haematology, University Medical Center Groningen, Hanzeplein 1, 9713 GZ Groningen, The Netherlands; 2 Department of Pharmacy, Apotheek Haagse Ziekenhuizen, The Hague, The Netherlands; 3 Department of Neurology, Medical Center Haaglanden, The Hague, The Netherlands

**Keywords:** Intracranial hemorrhage, Hemostasis, International normalized ratio, Prothrombin complex concentrate, Vitamin K antagonist

## Abstract

**Background:**

Millions of patients receive vitamin K antagonist (VKA) therapy worldwide. Annually 0.2–1 % of all VKA users develops an intracranial hemorrhage (ICH). Prothrombin complex concentrate (PCC) is administered to restore the INR ≤ 1.5 in an attempt to limit hematoma growth. In order to facilitate PCC dosing, our hospital recently changed from a variable dose based on bodyweight, baseline- and target-INR, to a fixed 1000 IU fIX PCC dosing protocol for ICH.

**Methods:**

In a before and after design, we compared successful achievement of an INR ≤ 1.5 with a fixed dosing strategy versus the variable dosing strategy of PCC in patients presenting with intracranial bleeding complications of VKA. Data of the two cohorts of patients were retrospectively collected from medical records.

**Results:**

A median dosage of 1750 IU was given per patient in the variable dose group (*n* = 25) versus 1000 IU in the fixed dose group (*n* = 28). In the intention-to-treat analysis, 96 % achieved an INR ≤ 1.5 after an initial dose in the variable dose cohort compared to 68 % in the fixed dose cohort (*p* = 0.01). An additional dose was given in 2 (8 %) versus 9 (32 %) patients, respectively (*p* = 0.04). The median door-to-PCC-order time was 42 versus 32 min (*p* = 0.37) and the door-to-needle time was 81, respectively 60 min (*p* = 0.42).

**Conclusion:**

The fixed dose protocol necessitates additional PCC infusions more frequently to achieve a target INR ≤ 1.5. Door-to-order and door-to-needle time were shorter but, in this small cohort, not significantly so. The effect on clinical outcome remains unknown.

## Introduction

Vitamin K antagonists (VKA) are used worldwide in primary and secondary prophylaxis of thromboembolic events. A major risk of VKA is the occurrence of intracranial hemorrhage (ICH). ICH is seen in 0.2–1.0 % of the anticoagulated population annually [1, 2]. Approximately 20 % of all ICH is associated with use of anticoagulants [1–3], with high mortality ranging from 40 to 60 % [4]. Rapid reversal of anticoagulant therapy in the management of ICH is crucial to reduce the risk of hematoma enlargement [5–7], which is in turn associated with poor outcome [8, 9].

Current American and European guidelines recommend administration of four-factor prothrombin complex concentrate (PCC) to reverse VKA therapy in ICH (level of evidence: ‘C: expert opinion’ and ‘very low’, respectively), however, without consensus regarding PCC dose calculation or the target international normalized ratio (INR) to be achieved [10, 11]. In terms of speed of administration, a fixed PCC dose instead of the conventional variable dose eliminates the need for dose calculation based on patient-specific variables. This is hypothesized to promote an earlier start of hemostasis restoration, favoring limitation of hematoma enlargement in ICH.

A hospital-wide implementation of a fixed PCC dosing regimen of 1000 IU fIX (factor IX), corresponding with 40 ml Cofact^®^ (Sanquin, Amsterdam), was introduced for VKA-associated bleeding complications in a Dutch teaching hospital. Several important arguments have advocated the use of a fixed dose: easier administration requiring no complex tables and input variables and reduction of door-to-needle time. Both factors are hypothesized to contribute to a better clinical outcome [7].

In the present study, we retrospectively compared achievement of the target INR (≤1.5) of the fixed dosing strategy versus the variable dosing strategy of PCC in patients presenting with a VKA-associated ICH. As timely management of ICH and consequently rapid reversal of anticoagulation are crucial in outcome in ICH, the door-to-needle time and time to achievement of target INR were of special interest and are included as secondary endpoints.

## Patients and Methods

### Overall Study Design

This is a retrospective study comparing the effect of two different PCC dosing strategies in a before and after design. The setting is a large teaching hospital that serves as regional neuro-trauma center. Here, a change in dosing protocol for PCC in VKA-related bleeding emergencies was introduced in November 2013. Before this date, the manufacturer’s recommended variable dose was used for PCC in VKA-related ICH. From November 2013, the variable dose was replaced by a fixed PCC dose for this indication.

The local Medical Ethics Committee reviewed the study protocol prior to data collection (METC Zuid Holland West, No. 14-061) and decided that, according to Dutch law, full review and informed consent were not required for this retrospective study.

### Patient Population

Patients were eligible for inclusion if they were at least 18 years of age and presented with ICH (primary or trauma-related), while on VKA therapy, for which PCC was administered. The inclusion time was from 1 January 2013 to 1 August 2014. November 2013 was considered as a protocol transition month, and therefore all eligible patients treated in this month were excluded. Patients were only included once in this study.

### Study Treatment

Until November 2013, patients were treated with a variable dose of PCC according to manufacturer’s specifications. This comprised a dose calculation based on patient bodyweight, initial INR and desired target INR (≤1.5). If target INR was not achieved when follow-up INR was assessed, additional dosing was left to the treating physicians discretion. After the change of protocol, patients were treated with a fixed dose of 1000 IU fIX PCC, followed by follow-up INR-assessment. If the target INR of ≤1.5 was not achieved, another 500 IU fIX PCC was administered.

A single brand of PCC was used within this timeframe, Cofact^®^ (Sanquin BV, Amsterdam, the Netherlands). This product contains factors II, VII, IX and X, as well as protein C and S without containing activated factors or heparin.

### Study Outcomes

The primary endpoint of the study was achievement of INR ≤ 1.5. Secondary end points included the time between presentation at the emergency department and the PCC order (door-to-order time), the time between entry into the emergency department and start of PCC infusion (door-to-needle time), the proportion of patients that needed an additional dose of PCC, the median INR after initial PCC administration, degree of disability according to the modified Rankin Scale (mRS) at discharge, duration of stay at the emergency department, duration of intensive care/stroke care unit stay, total duration of hospital stay, in-hospital thromboembolic or rebleeding events and mortality in-hospital and at 30 days after PCC administration.

### Data Collection

Both digital medical records from the hospital information system and paper medical records were retrieved for data collection. Data was collected regarding baseline characteristics (age, gender, weight, baseline INR, type of VKA and ICH type), primary outcome (follow-up INR after PCC administration up until 6 h after start of infusion or before additional PCC dose, whichever was first) and secondary outcomes.

Two authors (RA and IM) classified modified Rankin Score at discharge independently and reached consensus in case of disagreement, using reports in medical records and blinded from treatment regimen. Mortality status at 30 days after PCC administration was collected via general practitioners or nursing home doctors if this could not be obtained from the hospital information system.

### Statistical Analyses

Statistical analysis was performed using SPSS 22.0 (IBM Corporation, New York). All data were analyzed according to the intention-to-treat principle and findings were subsequently verified by performing a per-protocol analysis. For all outcome parameters, quantitative data was analyzed with Mann–Whitney *U* tests and categorical data with Fisher’s exact tests or Chi square tests.

## Results

**Fig. 1:**
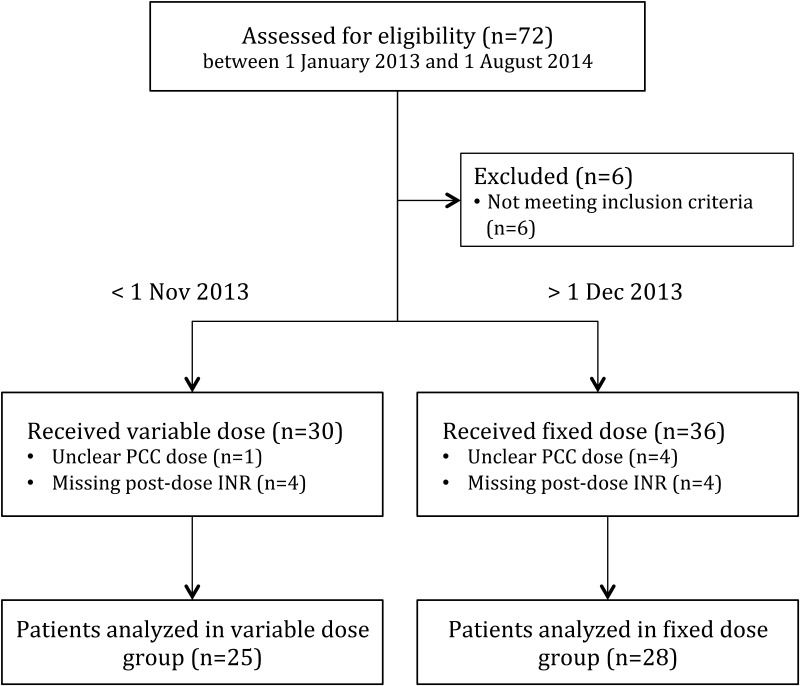
Flow chart of patient inclusion. Seventy-two patients received PCC for treatment of a VKA-associated ICH between January 2013 and August 2014. Six patients that presented with ICH in November 2013 were excluded, as this was the protocol transition month. 30 eligible patients remained in the group before November 2013 and 36 patients after. In both cohorts 4 patients were excluded due to a missing follow-up INR (lost to follow-up), while PCC dose could not be retrieved for 1 patient before versus 4 patients after November 2013. This resulted in 25 inclusions in the variable dose group and 28 inclusions in the fixed dose group

A total of 72 patients were considered for this study of which 53 met all inclusion criteria. Of these, 25 patients were included in the variable dose group and 28 patients in fixed dose group, as is seen in Fig. 1. Baseline characteristics are specified in Table 1. Both groups had similar baseline characteristics. The median age was 77 years and more than 70 % of the patients received VKA as part of atrial fibrillation therapy. The bleeding was intraparenchymal in approximately half of the patients; cause of bleeding was in 40 % of the patients related to trauma versus spontaneous bleeds in 60 %. Median baseline INR was also equivalent in both groups.

**Table 1:** Baseline characteristics of the variable and fixed dose group

	Variable dose group	Fixed dose group	*p* value
	*n* = 25	*n* = 28	
Gender (female); *n* (%)	12 (48 %)	14 (50 %)	1.000
Age (years); median (min–max)	77 (50–91)	77 (52–94)	1.000
Weight (kg); median (min–max)	79 (61–139)	73 (45–176)	0.211
INR; median (min–max)	3.1 (1.8–9)	3.3 (1.7–9)	0.175
VKA type			
Acenocoumarol*; n* (%)	6 (24 %)	5 (18 %)	0.737
Phenprocoumon; *n* (%)	19 (76 %)	23 (82 %)	
VKA indication			
Arrhythmia; *n* (%)	18 (72 %)	23 (82 %)	0.485
Deep vein thrombosis (prophylaxis); *n* (%)	7 (28 %)	4 (15 %)	
Artificial heart valve; *n* (%)	0 (0 %)	1 (4 %)	
ICH cause (traumatic); *n* (%)	11 (39 %)	10 (40 %)	1.000
ICH type			
IPH; *n* (%)	11 (44 %)	16 (57 %)	0.562
SDH; *n* (%)	8 (32 %)	4 (14 %)	
SAH; *n* (%)	4 (16 %)	3 (11 %)	
Other; *n* (%)	2 (8 %)	5 (18 %)	

*INR* international normalized ratio, *VKA* vitamin K antagonist, *ICH* intracranial hemorrhage; *IPH* intraparenchymal hemorrhage, *SDH* subdural hemorrhage, *SAH* subarachnoid hemorrhage

**Fig. 2:**
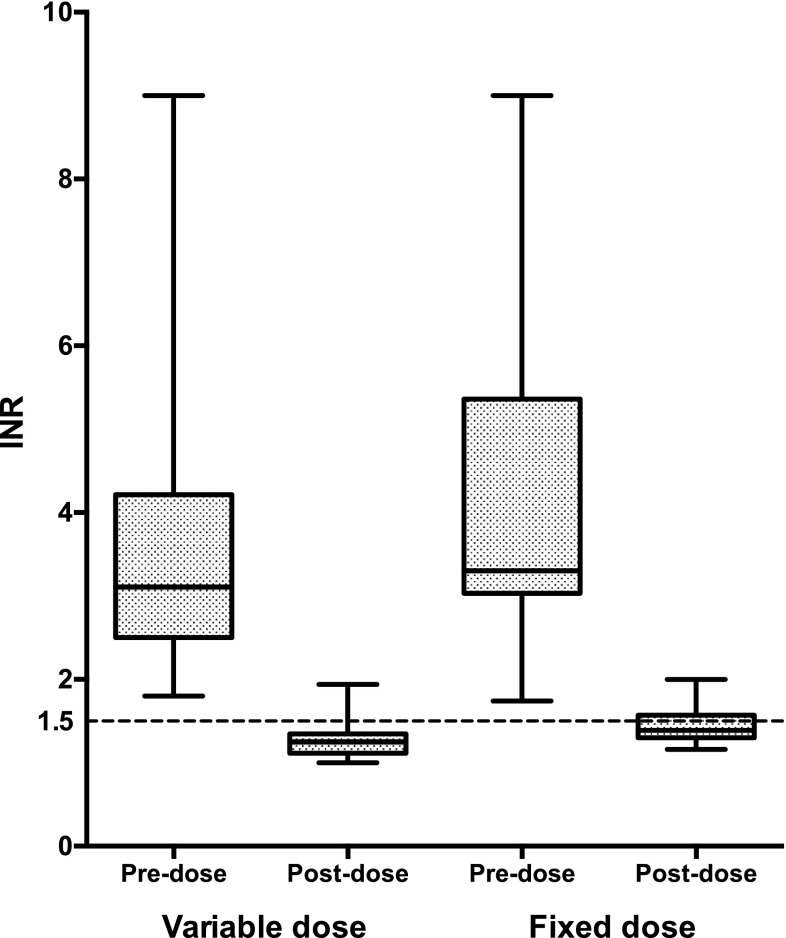
INR before and after initial PCC dose in the variable and fixed dose group. *Boxes* span the interquartile range with median, minimum and maximum values indicated by *horizontal bars*. The *dashed line* visualizes the achievement of target-INR ≤ 1.5

Successful achievement of INR ≤ 1.5 after the initial PCC dose was seen in 96 % of the patients in the variable dose group versus 68 % of the patients in the fixed dose group (Fig. 2). Median follow-up INR was significantly higher in the fixed dose group as compared to the variable dose group. Patients in the variable dose group received a median initial dose of 1750 IU, in the fixed dose group, 1000 IU (see Table 2) fIX. Two patients (8 %) (of which one had already achieved the target INR) received an additional dose in the variable dose group, versus nine patients (32 %) in the fixed dose group. The median total PCC dose did not differ from the median initial dose because more than half of the patients in each group reached the target INR with the initial dose.

**Table 2:** Results on primary outcome of both variable and fixed dose group

	Variable dose group (*n* = 25)	Fixed dose group (*n* = 28)	*p* value
INR ≤ 1.5 after initial PCC dose; *n* (%)	24 (96 %)	19 (68 %)	0.013
Additional dose received; *n* (%)	2 (8 %)	9 (32 %)	0.043
INR after initial dose; median (min–max)	1.3 (1–1.9)	1.4 (1.2–2)	0.001
Initial PCC dose (IU); median (min–max)	1750 (1000–2500)	1000 (1000–2500)	0.000
Total PCC dose (IU); median (min–max)	1750 (1000–2500)	1000 (1000–3000)	0.005

*INR* international normalized ratio, *PCC* prothrombin complex concentrate

There was no significant difference in time to treatment between the variable and fixed dose groups in door-to-order or door-to-needle time (Table 3). Data about mRS, mortality and duration of stay is also presented in Table 3. No rebleeding events were registered in both groups. Two patients in the variable dose group (8.3 %) developed a thromboembolic event characterized by worsening of hemiparesis on day 2 and day 4 after PCC administration. Subsequent CT-scans confirmed recent ischemia. The patients had a baseline INR of 2.8 and 3.6, which after PCC administration both reduced to 1.3. No thromboembolic events were seen in the fixed dose group.

**Table 3:** Results on secondary outcome parameters for both variable and fixed dose group

	Variable dose group	Fixed dose group	*p* value
	(*n* = 25)	(*n* = 28)	
Door-to-order time (min); median (min–max)	42 (0–207)	32 (3–211)	0.370
Door-to-needle time (min); median (min–max)	81 (33–231)	60 (24–251)	0.420
Modified Rankin Score ≤3 at discharge; *n*/total (%)	11/21 (52 %)	10/23 (44 %)	0.773
Mortality			
At discharge; *n* (%)	3 (12 %)	6 (22 %)	0.474
At 30 days after PCC treatment; *n* (%)	4 (16 %)	7 (25 %)	0.509
Duration of stay			
Emergency department (h); median (min–max)	2.7 (0–4.8)	3 (0–7.4)	0.232
Intensive/stroke care unit (days); median (min–max)	1.0 (0–17)	2.0 (0–14)	0.088
Total stay (days); median (min–max)	8 (1–43)	11.5 (1–52)	0.643

Mortality status at discharge and at 30 days after PCC treatment was known for all included patients. Door-to-order and door-to-needle time was unknown for 3 patients in the variable dose group and for 3 respectively 5 patients in the fixed dose group

Post hoc, we performed a subgroup analysis of patients with a baseline INR ≤ 4. In the variable dose group 19 out of 19 patients (100 %) with a baseline INR below 4 achieved the target INR, compared to 16 out of 18 (89 %) patients in the fixed dose group (*p* = 0.230). Median initial PCC dose was 1750 IU per patient in the variable dose group versus 1000 IU in the fixed dose group (*p* = 0.002).

## Discussion

This retrospective study shows that an initial fixed dose of 1000 IU fIX PCC is inferior in achieving the target INR of ≤1.5 in comparison to the variable dose calculated from bodyweight, baseline INR and target INR.

The choice of a fixed dose of 1000 IU might have been too low, as the target-INR achievement rate of the initial dose was only 68 %. Assuming an average bodyweight of 75 kg in our population, a median dose of 13 IU/kg can be calculated in the fixed dose group. In comparison, a previous French prospective observational multicenter study in ICH reported the use of a variable dose of 25 IU/kg, leading to an achievement rate of 76.5 % [12]. In contrast, another French prospective, but randomized, multicenter study in ICH found 100 % achievement in both 25 and 40 IU/kg treatment arms [13]. Our variable dose group demonstrated 96 % achievement with a median dose of 23 IU/kg bodyweight. Therefore, a higher initial dose in a fixed dose regimen might lead to achievement rates comparable to those found in variable dose regimens.

When restricting to patients with a baseline INR ≤ 4.0, post hoc analysis showed no significant difference between target INR achievement rates of variable dose and fixed dose groups. These achievement rates were acquired with a large between-group difference of 750 IU in median required PCC dose. Importantly, this has to be interpreted with caution, as it was a post hoc analysis using a small subgroup.

We found shorter, but not statistically significant so, door-to-order and door-to-needle times for the fixed dose group. As this study was not powered to demonstrate a difference, this could very well be a power issue requiring further investigation. In comparison, a significant and clinically relevant reduction of door-to-needle time was observed in a previous prospective cohort of extracranial bleeds when using a fixed dose. Median time to infusion reduced from 160 min in the variable dose group to 130 min in the fixed dose group. Although follow-up INR was higher after an initial dose as compared to the variable dose, clinical outcome was non-inferior or even better in the fixed dose group [14]. It was therefore hypothesized that a shorter door-to-needle time could be related to a comparable of even better clinical outcome.

Data on clinical outcome in our set of patients was however not recorded systematically trough functional outcome scales [i.e., modified Rankin Scale (mRS), Barthel Index (BI)]. mRS at discharge was assessable retrospectively from discharge reports. However follow-up mRS could not be determined because a large number of patients had no records on follow-up visits. Glasgow coma scores were recorded systematically, but frequently clouded by missing verbal scores due to aphasia and/or intubation. These impairments on verbal communication are inherent to ICH and lead to coma scores unrepresentative of the actual coma grade. In future (prospective) studies on this subject, data should be collected accordingly.

The strength of this study is that it used real life data on fixed dosing of PCC in ICH. Furthermore, the data of PCC dosing in ICH was well documented, therefore contributing to the quality of the study. This study also has important limitations. A main limitation is the small sample size, therefore lacking power to draw conclusions from data of the secondary endpoints. The sample size was however sufficient to demonstrate a significant difference in the primary outcome, achievement of target-INR. Another limitation is the retrospective nature of our study, resulting in the absence of parameters necessary for comparison of clinical outcome, such as follow-up CT scan, stroke classification according to the NIH Stroke Scale, BI and follow-up mRS scores.

Based on the findings of this first small retrospective study, implementation of a fixed dose of 1000 IU fIX cannot be recommended for VKA-related ICH. A fixed dose does have important advantages, compared to variable dose regimens, but 1000 IU is probably too low. Subsequent studies should include systematic evaluation of clinical outcome, in addition to INR results.

## Conclusion

By applying a fixed PCC dose (1000 IU fIX) target-INR was achieved in significantly less patients compared to a weight and INR-based PCC dose regimen for treatment of VKA-related ICH. Consequently, the fixed dose protocol necessitated additional PCC dosing to obtain the target-INR more frequently. The time between door-to-order and door-to-needle was not significantly shorter with the fixed dose protocol. The effect of PCC dose and target-INR on clinical outcome remains unclear.
